# Proteomics and transcriptomics of the BABA-induced resistance response in potato using a novel functional annotation approach

**DOI:** 10.1186/1471-2164-15-315

**Published:** 2014-04-28

**Authors:** Therese Bengtsson, Deborah Weighill, Estelle Proux-Wéra, Fredrik Levander, Svante Resjö, Dharani Dhar Burra, Laith Ibrahim Moushib, Pete E Hedley, Erland Liljeroth, Dan Jacobson, Erik Alexandersson, Erik Andreasson

**Affiliations:** 1 Department of Plant Protection Biology, Swedish University of Agricultural Sciences, Box 102, SE-230 53 Alnarp, Sweden; 2 Institute for Wine Biotechnology, Department of Viticulture and Oenology, Stellenbosch University, Stellenbosch, South Africa; 3 Department of Immunotechnology, Lund University, Lund, Sweden; 4 Department of Biology, Lund University, Lund, Sweden; 5 Genome Technology, James Hutton Institute, Invergowrie, Dundee, Scotland

**Keywords:** Functional annotation, Mevalonate pathway, *Phytophthora infestans*, Secretome, *Solanum tuberosum*, Sterol biosynthesis

## Abstract

**Background:**

Induced resistance (IR) can be part of a sustainable plant protection strategy against important plant diseases. β-aminobutyric acid (BABA) can induce resistance in a wide range of plants against several types of pathogens, including potato infected with *Phytophthora infestans*. However, the molecular mechanisms behind this are unclear and seem to be dependent on the system studied. To elucidate the defence responses activated by BABA in potato, a genome-wide transcript microarray analysis in combination with label-free quantitative proteomics analysis of the apoplast secretome were performed two days after treatment of the leaf canopy with BABA at two concentrations, 1 and 10 mM.

**Results:**

Over 5000 transcripts were differentially expressed and over 90 secretome proteins changed in abundance indicating a massive activation of defence mechanisms with 10 mM BABA, the concentration effective against late blight disease. To aid analysis, we present a more comprehensive functional annotation of the microarray probes and gene models by retrieving information from orthologous gene families across 26 sequenced plant genomes. The new annotation provided GO terms to 8616 previously un-annotated probes.

**Conclusions:**

BABA at 10 mM affected several processes related to plant hormones and amino acid metabolism. A major accumulation of PR proteins was also evident, and in the mevalonate pathway, genes involved in sterol biosynthesis were down-regulated, whereas several enzymes involved in the sesquiterpene phytoalexin biosynthesis were up-regulated. Interestingly, abscisic acid (ABA) responsive genes were not as clearly regulated by BABA in potato as previously reported in *Arabidopsis.* Together these findings provide candidates and markers for improved resistance in potato, one of the most important crops in the world.

## Background

Potato is today the third largest food crop in the world. One of the largest threats to potato production is late blight disease, caused by *Phytophthora infestans*, which is currently controlled by frequent use of fungicides. Extensive efforts to find more efficient weapons against late blight are ongoing worldwide. One alternative could be integration of induced resistance (IR) in current management strategies. IR, i.e. exposing plants to abiotic or biotic stress leading to improved resistance to subsequent pathogen attack both locally and systemically [1], is thought to function by activating or enhancing defence signalling [2]. This signalling is suggested to depend either on salicylic acid (SA) or jasmonic acid/ethylene (JA/ET) for the two classical forms of IR: systemic acquired resistance (SAR) and induced systemic resistance (ISR), respectively [3]. Resistance can be induced by treatment of plants with the xenobiotic amino-acid derivative dL-β-aminobutyric acid (BABA) [4,5]. BABA can enhance resistance in a wide range of plants against several types of pathogens, e.g. treatment increases the resistance of potato against *P. infestans*[4].

We have earlier shown by microscopy that a direct activation of defence responses associated to the formation of HR-like lesions takes place at efficient BABA-IR in the potato *Phytophthora* system [6]. This is in contrast to a “priming effect” proposed in other plant-pathogen systems [7]. The molecular mechanisms behind BABA-IR remains unclear and a better understanding on the molecular level could lead to the identification of biomarkers which in the future can be implemented in plant protection or breeding strategies.

Two microarray studies investigating BABA-IR have been conducted in *Arabidopsis*. Tsai et al., 2011 [8], reported that SA-responsive genes were both directly up-regulated by BABA treatment and up-regulated after priming by BABA and subsequent *Pseudomonas syringae* pv. *tomato* DC3000 infection. They also showed that BABA inhibits the *Arabidopsis* response to the bacterial phytotoxin coronatine, thus suppressing the coronatine-associated JA responses. The second microarray study of BABA-IR in *Arabidopsis* conducted by Zimmerli et al., 2008 [9] showed that BABA priming for increased thermotolerance involved ABA-associated transcription factors. They also showed that neither ET nor SA played a part in the acquired thermotolerance. These two microarray studies in *Arabidopsis* demonstrate the complexity of BABA-IR mechanisms. Furthermore, BABA has been shown to reduce vegetative growth and to cause major alterations in plant amino acid balance in *Arabidopsis*, effects that were reversed by L-glutamine treatment [10,11]. Based on these results, it was suggested that BABA provokes a stress-induced morphogenic response (SIMR) in *Arabidopsis* by activation of a general amino acid inhibition response. Another report indicates that the lectin-receptor kinase VI.2 is required for BABA priming of the PTI response and for BABA-IR against *Pseudomonas syringae* DC3000 in *Arabidopsis *[12].

In potato, a very important host for many pathogens such as *P. infestans,* the only previous RNA study relating to BABA was a time-series study using cDNA-AFLP [13]. Around 60 potato transcripts were found to be differentially expressed after BABA treatment and a clear overlap of genes affected by BABA and *Phytophthora* inoculation was found. Studies in transgenic NahG expressing potato plants, with an impaired SA metabolism, indicate that BABA-IR against *P. infestans* is dependent on SA [14]. BABA has also been shown to cause a direct activation of basal defence responses such as reactive oxygen species, hypersensitive response-like spots, phenolics and PR-1 expression in potato [6]. To elucidate molecular defence responses activated by BABA in potato, a genome-wide microarray analysis based on the recently released *Solanum tuberosum* group phureja genome [15] was performed in combination with label-free quantitative proteomic analysis of the apoplast secretome. Earlier reports showed that mRNA regulation in mammalian cells only explains about 40% of the changes in protein abundance [16] and the apoplast is a major interaction arena between plants and pathogens.

Genome-wide analysis is often hampered by incomplete and outdated functional annotations of genes and derived microarray probes. To this end, we present a network-based approach to retrieve information from orthologous gene families across 26 sequenced plant genomes, which increased the number of annotated probes by close to 9000.

## Methods

### Plant material and growing conditions

Potato plants (cv. Desiree) were grown under controlled conditions with 16 h/8 h day and night regime with a fluorescent lamp light intensity of 200 μmol m^-2^ s^-1^. The temperature was set to 20°C and the relative humidity was 65%. Pots were circulated every week to avoid positional effects. All sampling was done before flowering and only fully expanded leaves from the middle part of the plants were used. Plants were treated with BABA or water 2 days before harvest. For the treatments plants were randomly chosen, labeled and then placed back in the growth chamber evenly dispersed between treatments. For the detached leaf assays we used 29-day-old plants and for the molecular analysis we used 35-day-old plants.

### BABA treatment

Plants were treated with BABA (DL-3-aminobutyric acid, Sigma) dissolved in water at a concentration of 1.0 or 10 mM. BABA was sprayed on the plants until saturation (ca 40 mL) and control plants were sprayed with water as earlier described [6].

### *Phytophthora* inoculation and measurement

Four detached leaflets from each of three Desiree plants were infected with *P. infestans* two days after treatment with either 10 mM BABA or water (control)*. P. infestans* strain SE03058 isolated in mid-Sweden (mating type A1, virulence 1, 2, 3, 4, 7, 10, 11) was used with a sporangial concentration of 36,000 sporangia ml^-1^. The inoculated leaflets were kept in the dark at 15°C during the first day. On the second day the temperature was changed to 20°C with 16 h light/8 h dark. The size of lesion diameter was measured 7 days post inoculation (dpi). Data from two separate experiments were combined for a total of 18 biological samples. The difference in lesion sizes between the treatments were analysed by Student’s *t*-test with Minitab v.16.

### Gene ontology annotation and enrichment analysis

Genome annotation is commonly done by orthologous inference, the transfer of annotation across genomes based on orthologous relationships between genes in different genomes. There are a number of different algorithms available to determine orthologous relationships [17]. OrthoMCL is an orthology detection method which clusters genes into orthologous gene families based on sequence similarity [18]. However, the OrthoMCL software can currently not be applied to large datasets. Thus a parallel version of OrthoMCL (Parallel-OrthoMCL) was developed in house in order to allow the OrthoMCL algorithm to be applied to large datasets. Orthologous gene families across 26 plant species were constructed using Parallel-OrthoMCL. 25 plant species available from PLAZA [19] were included in the analysis and the protein translations of the longest representative transcript from the *Solanum tuberosum* group phureja genome was downloaded from the Solanaceae Genomics Resource [ftp://ftp.plantbiology.msu.edu/pub/data/SGR/GO_annotations/]. The OrthoMCL percent match parameter was set to 50% and the MCL inflation value was set to 2.0. These are values commonly used by other genome annotation pipelines such as PLAZA [19]. Once gene families had been constructed, potato genes were annotated using a network-based annotation as follows: (i) all available GO annotations in PLAZA were mapped onto the appropriate genes in each of the 25 species contained in PLAZA; (ii) each potato gene was then assigned the GO terms of all the other genes from the 25 other species that were present in its specific gene family: (iii) the probes from the potato microarray (Agilent JHI *Solanum tuberosum* 60 k v1) were then annotated by assigning to each probe the GO terms of the genes corresponding to that probe as determined by BLASTing the probe sequences against the nucleotide transcript sequences from the potato genome. The probes of the potato microarray were also annotated using the potato gene GO annotation file downloaded from the Solanaceae Genomics Resource (SGR annotation). As described above, probes were annotated by assigning each probe the GO terms of the genes corresponding to that probe. The two microarray probe annotations were compared to identify the number of probes annotated in each annotation and also to identify the distribution of the number of GO terms assigned to each probe.

### RNA sample preparation and microarray analysis

In total one leaflet from each of three plants per treatment was used for RNA analysis. Five leaf discs of 10 mm diameter were punched out of each leaflet, pooled, and immediately frozen in liquid nitrogen. The frozen samples were homogenized for 30 s at 30 rpm in a Retsch Mixer Mill MM 200 (Retsch GmbH, Haan, Germany) followed by extraction of RNA, DNase treatment and purification using RNeasy Mini kit (Qiagen GmbH, Hilden, Germany). RNA concentration and purity (260/280 nm >1.8) was checked by a ND-1000 NanoDrop (Wilmington, USA) and integrity of the samples were analyzed with an Experion™ Automated Electrophoresis System (Bio-Rad Laboratories, Hercules, USA). Each sample was diluted in RNase-free water to a concentration of 200 ng/μl and mRNA expression analysis was done with a custom-made Agilent expression array based on the predicted transcripts of the potato genome (version 3.4). The complete microarray design is available in ArrayExpress (E-MTAB-1655). The arrays were run according to the instructions and recommendations of the supplier (Agilent). The probe intensities were background corrected and normalised using the quantile method in the Limma R-package [20]. Fold changes and standard errors were obtained by fitting a linear model to each gene expression values and standard errors were smoothed based on the empirical Bayes method. Genes with p-values below 0.05 after adjustment (Benjamini-Hochberg) were regarded as significant. The microarray data was deposited in ArrayExpress, accession number: E-MTAB-1545. Significantly differentially expressed genes were analyzed for Enrichment of Gene Ontology Terms in GOEast using default settings on the basis of a custom made GO enrichment list for the microarray. To give an overview of all significantly enriched GO terms ReviGO was applied to cluster terms based on semantics [21]. Based on the potato genome (version 3.4) and manual curation, genes and proteins were functionally characterized by the MapMan mapping file.

### Secretome extraction and visualisation

Two days after BABA treatment, three biological replicates in the form of separate plants were subjected to analysis. Four leaflets per plant were harvested on ice. Secretome proteins were extracted by vacuum infiltration with a phosphate buffer directly after harvest followed by centrifugation as described by Ali et al., 2012 [22] and Alexandersson et al., 2013 [23]. For visualisation of the apoplastic proteins, 30 μl of the samples were separated on 14% SDS polyacrylamide gels. The samples were mixed with 6 × SDS sample buffer containing DTT and denatured at 93°C for 3 min prior to electrophoresis. The gels were run for 20 min at 80 V followed by 1.5 h at 100 V and staining overnight in Coomassie Brilliant Blue (10% (v/v) acetic acid, 45% (v/v) methanol, and 0.25% (w/v) Coomassie R-250). Finally, the gels were briefly washed in dH_2_O before 3 h in destaining solution (10% (v/v) acetic acid and 40% (v/v) methanol).

### Identification and quantification of proteins by LC-MS/MS

For identification of the apoplastic proteins, 30 μl of the secretome samples were separated with SDS-PAGE as described for the visualisation of proteins, but the gel was only run 10 mm. The gels were stained with Coomassie Brilliant Blue and the stained portions cut into 1–2 mm^2^ pieces. Tryptic digestion of the gel pieces and peptide elution was carried out as described by Ali et al., 2012 [22]. The eluted peptides were concentrated to 50 μl by rotary evaporation and stored at -80°C. Six μl of sample was subjected to HPLC-MSMS analysis using an Eksigent nanoLC2D HPLC system coupled to an Orbitrap XL. The peptides were loaded onto a pre-column (Agilent Zorbax 300SB-C18, 0.3 mm ID, 5 mm, 5 μm particle size) connected to an analytical column (Agilent Zorbax 300SB C18, 75 μm ID, 150 mm, 3.5 μm particle size). The analytical column was pre-equilibrated for 10 min using buffer consisting of 0.1% formic acid (FA), 5% acetonitrile (ACN) at a flow rate of 10 μl/min, and the peptides were separated in an 0.1 FA buffer using a 55 min linear gradient from 5% to 40% ACN followed by a 5 min linear gradient from 40% to 80% ACN at a flow rate of 350 nl/min. The eluted peptides were analysed using an LTQ Orbitrap, operated in data-dependent mode to automatically perform Orbitrap-MS and LTQ-MSMS analysis. Survey scan spectra (400–2000 Da) were acquired using the Orbitrap mass analyzer with the resolution R = 60000. Automatic gain control was enabled. The seven most intense ions were selected for fragmentation in the LTQ, using a mass window of 2 Da for precursor ion selection. The precursor ions were fragmented with normalised collision energy of 35 (with activation Q set to 0.25 and an activation time of 30 ms). Dynamic exclusion was enabled with a repeat count of 2, a repeat duration of 20 seconds, exclusion duration of 120 seconds, an exclusion list size of 499 and a 10 ppm exclusion mass width relative to both low and high. This raw data was then converted to mgf files using ProteoWizard [24]. The Proteios software environment [25] was used to search the files with Mascot against a database consisting of the protein sequences from the potato genome and all *Solanaceae* sequences from UniProt extended with an equal size random part, with conserved protein length and amino acid distribution for the random part. Search tolerances were set to 5 ppm for MS and 0.5 Da for MS/MS. One missed cleavage was allowed. Carbamidomethylation of cysteine residues was selected as a fixed modification and oxidation of methionine residues was selected as a variable modification. Proteios was used to identify hits at an estimated peptide spectrum match false discovery rate (FDR) of 0.01 using the random database mentioned above. For quantitative analysis, a label-free approach based on precursor intensities was used [26], with msInspect feature detection [27], and Proteios alignment as described previously [28]. Quantitative data from different conditions was normalised using a linear transformation procedure where the intensity of each feature was divided by the sum of all features in the same run and then multiplied by the average of the summed intensities of all runs for that replicate. Missing values were set to 1. This data was then log-transformed and analysed using an ANOVA based approach in the Qlucore software (http://www.qlucore.se). Peptides that were differentially abundant were identified by performing a two-group comparison Qlucore with a FDR < 0.1 according to the Benjamini-Hochberg procedure for determining adjusted p-value, *q*. Only peptides uniquely identifying one protein in the potato genome (version 3.4) were used in order to avoid ambiguous peptides identifying several similar proteins.

## Results

### *P. infestans* growth after BABA treatment

**Figure 1:**
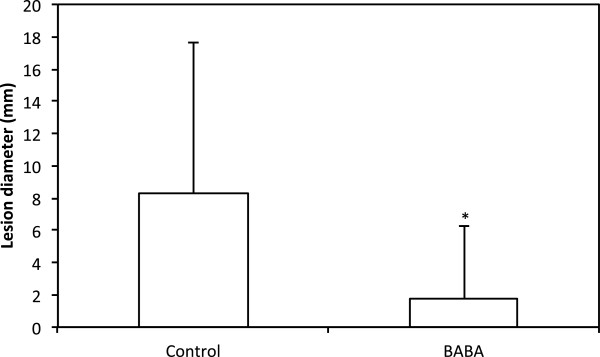
**Detached leaf assay.** Mean lesion sizes of *P. infestans* infection in potato leaves, 7 dpi. Leaflets (N = 18) were infected two days after treatment with BABA (10 mM) and water (control). Bars represent Standard Error of the mean. Treatment with a significant effect (p <0.05) is marked with *.

To find out if BABA at a concentration of 10 mM could restrict *P. infestans* growth in potato cv. Desiree, a detached leaf assay was performed. BABA led to significantly reduced lesion sizes of *P. infestans* in Desiree at 7 dpi (p = 0.012; Figure 1). Small necrotic lesions were visible on the leaves after treatment with BABA at 10 mM but not 1 mM (not shown). These were not observed in the controls, where leaves were treated with water. BABA at 1 mM was not tested since we earlier demonstrated a threshold level of 2.5 mM BABA for successful restriction of *P. infestans* growth in potato [29].

### New network-based annotation of potato genes

**Figure 2:**
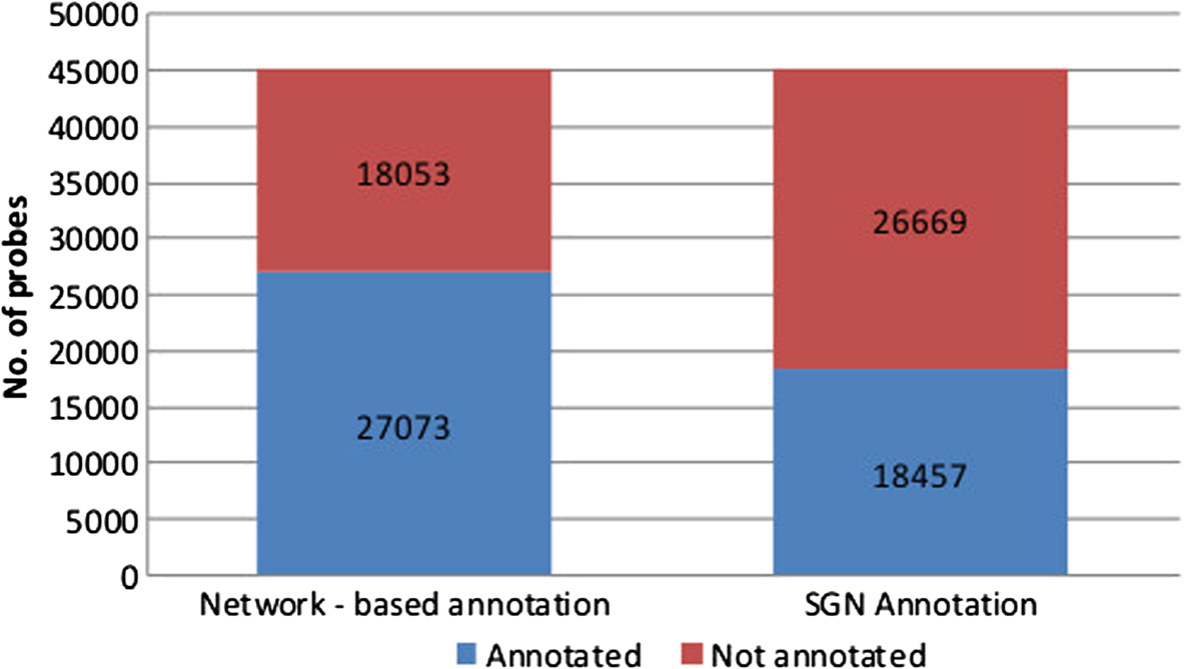
**Annotation Coverage.** A comparison of the number of annotated and un-annotated probes of the Agilent JHI *Solanum tuberosum* 60 k v1 microarray, using network based annotation (new annotation) and Solanaceae Genomics Resource (SGR) annotation.

Network-based annotation of the potato microarray probes and PGSC gene models applying parallelised OrthoMCL across 26 sequenced plant genomes was compared to the annotation of the potato microarray probes provided by Solanaceae Genomics Resource (SGR). Of the available ortholog detection algorithms, OrthoMCL has previously been shown to have the best performance (and the lowest false positive rate) in ortholog detection across multiple genomes [17]. As can be seen from Figure 2, 8616 additional probes out of a total of 45126 receive an annotation based on the 26 genomes with the SGR annotation. The number of ontology terms assigned to a probe is one of the indicators of the amount of information linked to the probe. In comparison to the earlier annotation, the number of GO terms assigned per probe was much larger with a median of 34 GO terms assigned per probe for the new annotation versus 6 for the SGR annotation (Additional file 1: Figure S1). Thus, the new annotation using gene families constructed from 26 plant genomes provides a more complete annotation of the potato microarray than was possible with the annotation file provided by the SGR.

### Overall transcript and protein changes

In the microarray analysis, 5378 transcripts were significantly (adj. p-value <0.05) differentially expressed in potato leaf 48 h after treatment with 10 mM BABA (Table 1). In the corresponding apoplast secretome samples, 91 proteins (50 up- and 41 down-regulated) were found to change in abundance (Table 1). BABA applied at the lower concentration (1 mM) had almost no influence on gene expression. Only six differentially expressed transcripts were identified (1 up- and 5 down-regulated), in contrast to 24 apoplastic proteins (11 up- and 13 down-regulated) with a significantly changed abundance (Tables 1 and 2). The only transcript up-regulated by 1 mM BABA, a MutT domain protein (G400017400), was also up-regulated by 10 mM BABA.

**Table 1:** Overview of BABA-induced changes

**BABA concentration**	**Gene expression**		**Secreted proteins**	
	**Up**	**Down**	**Up**	**Down**
1 mM	1	5	11	15
10 mM	3272	2106	50	42

Total number differentially expressed transcripts (adj. p-value <0.05) and proteins (FDR < 0.1) with altered abundance in leaves (cv. Desiree) treated with BABA (1 and 10 mM). Samples were analysed two days after treatment of the leaf canopy with BABA.

**Table 2:** Differentially expressed transcripts in potato leaves treated with 1 mM BABA

**Protein name**	**Gene id**	**Protein id**	**log2 Fold change**
**Protein phosphorylation**			
WAK-like kinase	G400010032	P400017740	-1.4
**Metabolic processes**			
MutT/nudix domain protein	G400017400	P400030392	3.5
Glucan/water dikinase	G400016613	P400029040	-1.0
**Unknown function**			
Hypothetical gene of unknown function	G400044248	P400066352	-1.6
Hypothetical gene of unknown function	G400031825	P400055091	-1.4
Uncharacterized mitochondrial protein AtMg00030	G400019855	P400034471	-1.3

Samples were analysed two days after treatment of the leaf canopy with BABA. Transcripts with significantly changed expression (adj. p-value <0.05) are included in the table.

The overlap of regulation found between the transcript and protein level was low. Of the 91 proteins differentially regulated by 10 mM BABA, only 13 overlapped at the transcript level. Ten out of these were up-regulated both at the transcript and protein levels, i.e. four endochitinases (G400033882, G400008796, G400008673, G400001528), three glucan endo-1,3-beta-glucosidases (G400029830, G402000722, G400010490), two peroxidases (G400012589, G400027614) and one DUF26 domain-containing protein 1 (G400041220). Interestingly, three genes were down-regulated at the protein level but up-regulated at the transcript level, namely pectin acetylesterase (PAE) (G014024140), serine-threonine protein kinase (G400016908) and xylem serine proteinase 1 (G400019147).

### Gene ontology analysis and gene expression after 10 mM BABA treatment

**Figure 3:**
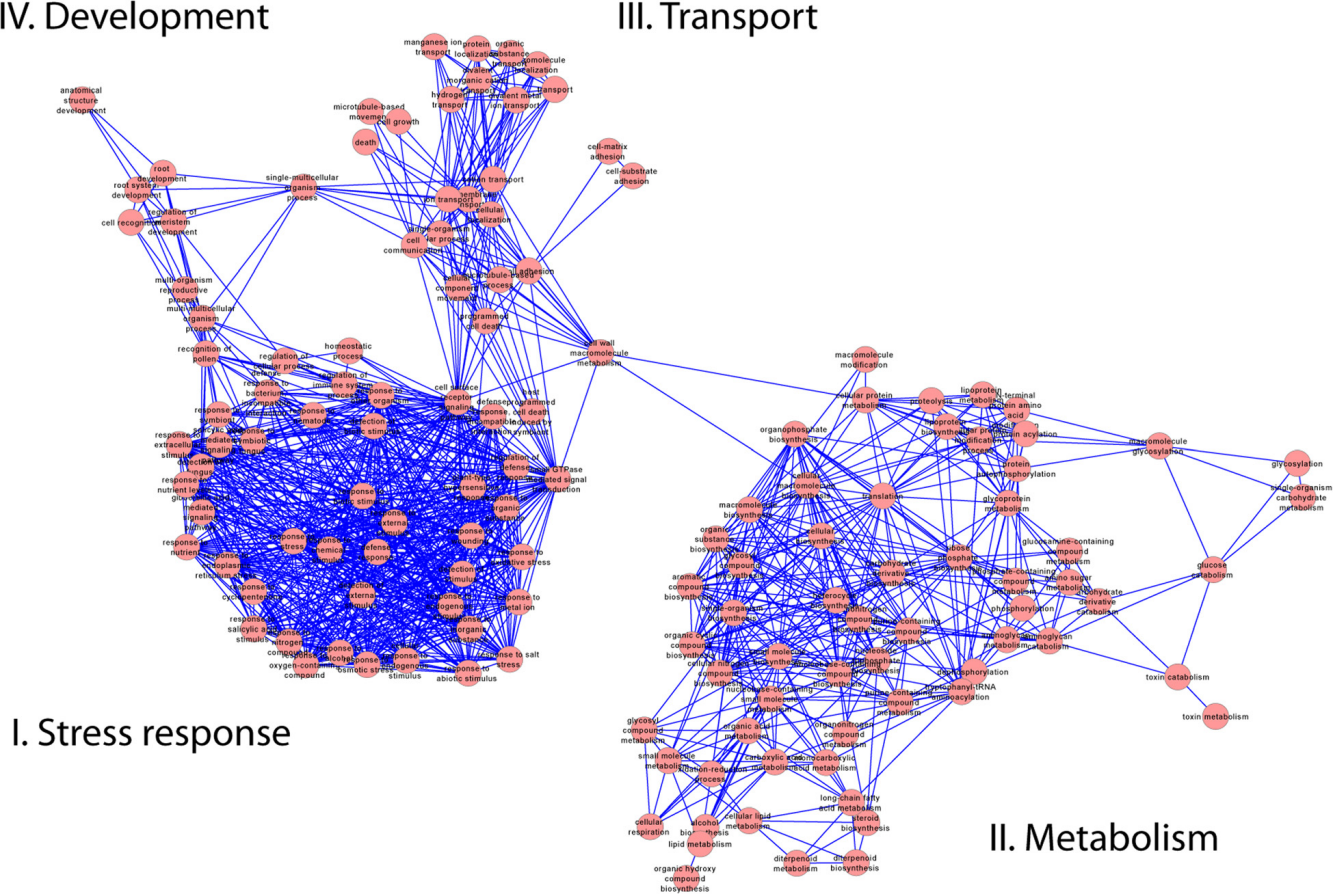
**ReviGO analysis of enriched GO terms.** Significantly enriched GO terms were clustered by semantic similarities and four overall clusters pointed out.

With more than 5000 transcripts affected by 10 mM BABA, it is evident that many functional mechanisms were altered. In order to identify processes where gene expressional changes occur, a gene ontology (GO) enrichment analysis was done based on the extended functional annotation described above. The 15 most enriched GO terms are described in Table 3. In all, a large number of GO terms (>500) were significantly enriched (p <0.1; Additional file 2: Table S1). To give an overview of all GO terms we used ReviGO, which clusters GO terms based on semantic similarities [21]. This analysis shows two major clusters of terms associated to stress response and metabolism as well as two minor clusters related to development and metabolism (Figure 3).

**Table 3:** Gene ontology enrichment

**GOID**	**Ontology**	**Term**	**p-value**
GO:0016023	Cellular_component	Cytoplasmic membrane-bounded vesicle	1.4E-48
GO:0014070	Biological_process	Response to organic cyclic compound	9.0E-32
GO:0004674	Molecular_function	Protein serine/threonine kinase activity	4.6E-25
GO:0006796	Biological_process	Phosphate-containing compound metabolic process	1.2E-24
GO:0006464	Biological_process	Cellular protein modification process	1.4E-24
GO:0036211	Biological_process	Protein modification process	1.4E-24
GO:0004672	Molecular_function	Protein kinase activity	2.9E-24
GO:0016773	Molecular_function	Phosphotransferase activity, alcohol group as acceptor	5.5E-24
GO:0016301	Molecular_function	Kinase activity	3.8E-23
GO:0009751	Biological_process	Response to salicylic acid stimulus	2.8E-22
GO:0044267	Biological_process	Cellular protein metabolic process	6.9E-22
GO:0006468	Biological_process	Protein phosphorylation	4.2E-18
GO:0016310	Biological_process	Phosphorylation	6.3E-18
GO:0010583	Biological_process	Response to cyclopentenone	1.2E-17
GO:0004713	Molecular_function	Protein tyrosine kinase activity	9.8E-17

Gene ontology results of microarray experiment run on leaves (cv. Desiree) two days after treatment of the leaf canopy with BABA (10 mM). The 15 most enriched GO terms are listed.

In Table 4, probes corresponding to the 50 most up- and down-regulated microarray transcripts were divided into functional categories. A full list of significant differentially expressed genes after 10 mM BABA treatment is included in Additional file 3: Table S2. Enriched GO terms after BABA treatment included processes associated with induction of plant defence such as plant-type hypersensitive response, necrotic spotted lesions, cell surface receptor signalling pathways, innate-immune response, defence response to fungus, and systemic acquired resistance (SAR). Examples of genes linked to these processes were two *NDR1* homologs (G400032148, G400032231), two *EDS1* splice variants (G400033029), two *phytoalexin-deficient 4–2 protein* splice variants (G400019873), several *ethylene receptor* splice variants (e.g. G400031819) and *PR1* homologs (e.g. G400005112). When categorising genes to cellular compartments one of the most enriched GO terms is cytoplasmic membrane-bound vesicle (Table 3). This category included KiTH-2 (G400008097- G400008100) and Wound-induced protein WIN1 (G400019435) similar to AtPR4, which were among the most up-regulated transcripts after BABA treatment (Table 4). Among these genes was also the potato *PEN1* (G400021331) homolog. WIN1 together with an Nb cell death marker (G400010131), osmotin (PR-5; G400003057), and Nectarin 5 (G400031325) were among the four most up-regulated transcripts involved in stress response. One Kunitz proteinase inhibitor and one serine protease inhibitor are among the most down-regulated transcripts related to stress response (G400009512, G400010128). There were no differentially expressed genes among the 17 genes annotated as R-genes on the array. However, the expression of some other LRR transcripts changed significantly. A TIR/NBS/LRR protein (G400001756) is up-regulated 8 times and three NBS-LRR type proteins (G400015872, G400027797, G400043650) are up- or down-regulated about two times. Only subtle expression differences were seen among other members in this large gene family.

**Table 4:** Differentially expressed transcripts in potato leaves treated with 10 mM BABA

**Protein name**	**Gene id**	**Protein id**	**log2 Fold change**
**Transport**			
Glucose-6-phosphate/phosphate translocator 2	G400025495	P400044203	5.2
P-rich protein EIG-I30	G400004797	P400008501	6.2
Tetrapyrrole synthesis			
Gamma aminobutyrate transaminase isoform 2	G400024281	P400042007	-3.0
**Stress**			
Nb cell death marker	G400010131	P400017936	5.5
Nectarin 5	G400031325	P400054574	5.2
Osmotin	G400003057	P400005490	5.5
Wound-induced protein WIN1	G400019435	P400033767	7.5
Chitin-binding lectin 1	G400019517	P400033919	-4.7
Kunitz-type proteinase inhibitor	G400009512	P400016823	-4.5
Major latex	G400028725	P400050037	-3.0
Major latex	G400028724	P400050036	-3.2
Serine protease inhibitor 7	G400010128	P400017933	-5.6
**Secondary metabolism**			
Tryptophan decarboxylase	G400018358	P400031995	5.1
Tryptophan decarboxylase	G400018359	P400031996	5.5
Linalool synthase	G401011252	P400019916	-3.0
Phenylcoumaran benzylic ether reductase	G400003691	P400006583	-3.9
**RNA**			
AT-HSFB3 (*Arabidopsis* thaliana heat shock transcription factor B3)	G401008167	P400014364	5.7
CHP-rich zinc finger protein	G400028967	P400050478	5.1
MYB8	G400020071	P400034798	-3.9
MYC1	G401010822	P400019120	-3.3
Zinc finger protein	G401014997	P400026368	-3.0
**Protein**			
E3 ubiquitin-protein ligase RMA1H1	G400021683	P400037599	-4.1
Phytophthora-inhibited protease 1	G400004380	P400007779	-3.5
Nucleotide metabolism			
MutT domain protein	G400017400	P400030392	7.8
**Lipid metabolism**			
Non-specific lipid-transfer protein	G400032250	P400055449	5.8
C-4 sterol methyl oxidase 2	G400002156	P400003856	-3.7
C-4 sterol methyl oxidase	G400012763	P400022612	-4.8
Non-specific lipid-transfer protein 2	G400011953	P400021168	-3.2
3-ketoacyl-CoA synthase	G400014549	P400025626	-3.4
**Hormone metabolism**			
Conserved gene of unknown function	G400001001	P400001896	5.7
Copalyl diphosphate synthase	G400001948	P400003472	5.5
Lipoxygenase	G400010859	P400019187	6.5
DWARF1/DIMINUTO	G400021142	P400036651	-4.8
ERF1	G400025989	P400045082	-4.9
Sterol delta-7 reductase DWF5	G400005931	P400010516	-3.4
**Development**			
KiTH-2	G400008097	P400014246	8.6
KiTH-2	G400008098	P400014247	6.4
KiTH-2	G400008099	P400014248	9.2
KiTH-2	G400008100	P400014249	6.3
Nodulin	G400030381	P400052889	-3.3
Patatin-2-Kuras 4	G400014104	P400024808	-5.5
Patatin-04/09	G402017090	P400029876	-5.3
Patatin group O	G400029247	P400050915	-5.0
**Cell wall**			
Glucan endo-1,3-beta-glucosidase, basic isoform 1	G400040260	P400062364	5.1
Major pollen allergen Ory s 1	G400026220	P400045511	5.7
Cellulose synthase	G400011752	P400020836	-4.9
Fasciclin-like arabinogalactan protein 19	G400017376	P400030347	-3.1
**Biodegradation of Xenobiotics**			
2-Hydroxyisoflavanone dehydratase	G400031849	P400055127	6.2
**Oxidation-reduction process**			
Cationic peroxidase	G400012589	P400022299	6.6
DC1 domain-containing protein	G401008889	P400015622	5.4
Polyphenol oxidase	G400018916	P400032951	6.2
Oxidoreductase, 2OG-Fe(II) oxygenase family protein	G402003479	P400006181	-5.1
Oxidoreductase, 2OG-Fe(II) oxygenase family protein	G400021383	P400037071	-4.3
Oxidoreductase, 2OG-Fe(II) oxygenase family protein	G400032208	P400055421	-5.2
**Not assigned/Misc**			
Ankyrin repeat-containing protein	G400013763	P400024321	6.1
Arachidonic acid-induced DEA1	G400039214	P400061318	5.5
ATP-binding component of a transport system	G400019445	P400033804	6.5
Citrate binding protein	G400003993	P400007118	7.3
Conserved gene of unknown function	G400024991	P400043372	5.3
Conserved gene of unknown function	G400031326	P400054575	5.6
Conserved gene of unknown function	G400002292	P400004083	6.5
Cytochrome P450	G400043512	P400065616	6.5
Gene of unknown function	G400025446	P400044135	5.6
Hypothetical repeat protein	G400028412	P400049430	5.2
Lichenase	G400020017	P400034720	5.2
P-rich protein EIG-I30	G400004737	P400008397	7.9
Primary amine oxidase	G400030082	P400052390	5.7
Abhydrolase domain containing	G400014029	P400024703	-4.0
Blue (Type 1) copper domain	G400013271	P400023469	-3.4
Conserved gene of unknown function	G400015196	P400026658	-3.0
Conserved gene of unknown function	G400015948	P400027933	-3.6
Cytochrome P-450	G400011750	P400020830	-3.7
Family 1 glycosyltransferase	G400027200	P400047278	-4.2
Gene of unknown function	G400040677	P400062781	-4.0
Glucosyltransferase	G400005960	P400010581	-3.2
Glucosyltransferase	G402027210	P400047294	-5.3
SGA	G400011740	P400020813	-5.7

The 50 up- or down-regulated transcripts with the highest- fold change (including splice variants, not shown in table) in potato leaves following treatment with 10 mM BABA. Samples were analysed two days after treatment of the leaf canopy with BABA. Transcripts with significantly changed expression (adj. p-value <0.05) are included in the table.

Significant enrichments were found for genes associated with the hormones ethylene, ABA, SA, JA and cyclopentenone, but of the processes related to hormone metabolism, only gibberellin biosynthesis was enriched. In this pathway, several splice variants of copalyl diphosphate synthases, which act early in biosynthesis of gibberellins, were sharply up-regulated (Additional file 2: Table S1 and Additional file 3: Table S2). Furthermore, several gibberellin 20-oxidases (G400036251, G400039968, G400040776, G400036003), which catalyze the conversion of different gibberellin forms, as well as gibberellin 2-oxidase 1, which is able to de-activate gibberellins [30], were down-regulated by BABA, except for one transcript (G400015796) that was slightly up-regulated. In addition, several gibberellic acid insensitive dwarf (GID) proteins (G400022493, G400022492, G401008665, G400018153, G400015446) which are GA-receptors regulating the stability of DELLA proteins, were also affected by BABA treatment, and two splice variants of DELLA proteins (GAI) (G400019212) were up-regulated.

In JA-regulation, two Jaz proteins were oppositely regulated, with transcripts representing *Jaz1* (G400002930) up-regulated and *Jaz3* (G400032119) down-regulated. Several splice variants of *MYC1* (G401010822) and *MYC2* (G404010822 G400005525, G402010822) transcription factors were down-regulated, whereas two splice variants of a JA-induced *WRKY* (G400019824) were up-regulated. JAZs act as negative regulators of the JA signalling pathway by binding to MYC transcription factors [31]. In JA biosynthesis (although not significant in the GO analysis), allene oxide cyclase (AOC) (G401012679), was down-regulated, whereas splice variants of lipoxygenase (LOX) (G400010859, G400007793) and Acyl-coenzyme A oxidase (G400010548, G400020620) were up-regulated.

Ethylene response factor 1 (ERF1) (G400025989), a positive regulator of JA and ET signalling [32], was down-regulated. However, two splice variants of 1-aminocyclopropane-1-carboxylate oxidase 2 (ACC) (G400013894), involved in the last step of ET biosynthesis, were up-regulated by 10 mM BABA.

In the brassinosteroid (BR) pathway, several brassinosteroid insensitive 1-associated receptor kinase 1 s (BAKs) were up-regulated. Among the 50 most down-regulated are transcripts encoding two forms of the DWARF1/DIMINUTO protein (G400021142) which are involved in early brassinosteroid biosynthesis. The *Arabidopsis* DWARF1 knock-out mutant has stunted growth and an altered secondary cell wall [33].

In the auxin pathway, TIR1 (G400020729) along with several auxin-response factors (ARFs) were down-regulated by BABA, but one ARF 5 (G400003771) was up-regulated. The transcript of auxin-induced SAUR (G400013321) was also up-regulated.

Treatment with 10 mM BABA also affected the regulation of several transcripts involved with amino acid metabolism e.g. related to proline, arginine and glutamine biosynthesis in the glutamate pathway. The most profound up-regulation was found in transcripts for biosynthesis of aromatic amino acids, e.g. chorismate mutase 1 (G400001438), arogenate dehydratase (G400007122) and arogenate dehydrogenase 1 (G400030683, G400020334) involved in phenylalanine and tyrosine biosynthesis [34]. Several transcripts of tryptophan decarboxylase (TDC) (G400018359, G400018358, G400018357, G400005444, G400035067) and one transcript of strictosidine synthase (G402017288) were highly up-regulated.

**Figure 4:**
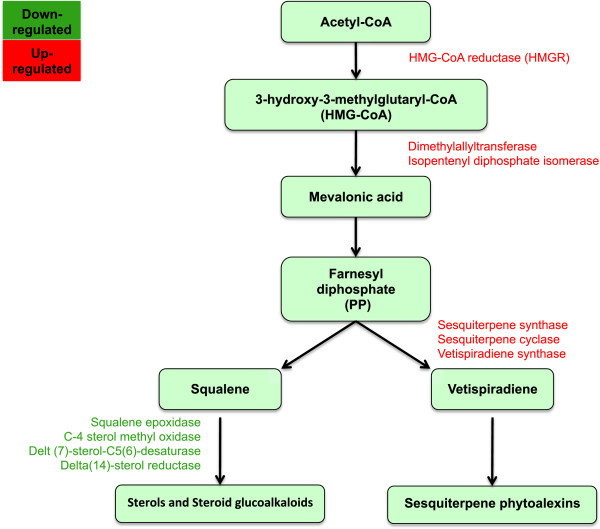
**BABA-influenced changes within the mevalonate pathway.** The effect of 10 mM BABA on transcripts encoding genes within the mevalonate pathway in leaves of potato. BABA caused a down-regulation of several transcripts involved with sterol biosynthesis, whereas transcripts involved with sesquiterpene phytoalexin biosynthesis were up-regulated. Transcripts with significantly changed expression (adj. p-value <0.05) are denoted in red (up-regulation) and green (down-regulation).

Two C-4 sterol methyl oxidase (SMO; G400002156, G400012763) transcripts were found among the 50 most down-regulated by 10 mM BABA (Table 4). Other enzyme-coding transcripts involved in sterol biosynthesis and glucosylation and in part of the mevalonate pathway, for example sterol delta-7 reductase DWF5 (G400014722, G400005931), DWARF1/DIMINUTO (G400021142), delta-7-sterol-C5(6)-desaturase (G400026401), squalene epoxidase (G400003324), delta-14-sterol reductase (G400002x720), and a UDP-glucose:sterol glucosyltransferase (G400015817), were also down-regulated. In contrast, transcripts encoding several sesquiterpene synthase 2 (G400010587, G400010585, G400010593, 400029200, G400029201, G400009854, G400018066, G400019782, G400009832) and vetispiradiene synthase 1 (G400018245), enzymes involved in sesquiterpene phytoalexin biosynthesis and belonging to another branch of the mevalonate pathway, were strongly up-regulated (Figure 4).

BABA also seemed to influence genes involved in cell wall structure, e.g., several cellulose synthase genes were slightly up-regulated whereas one was found among the 50 most down-regulated transcripts (Table 4).

### Secretome SDS-PAGE and LC-MS/MS analysis

**Figure 5:**
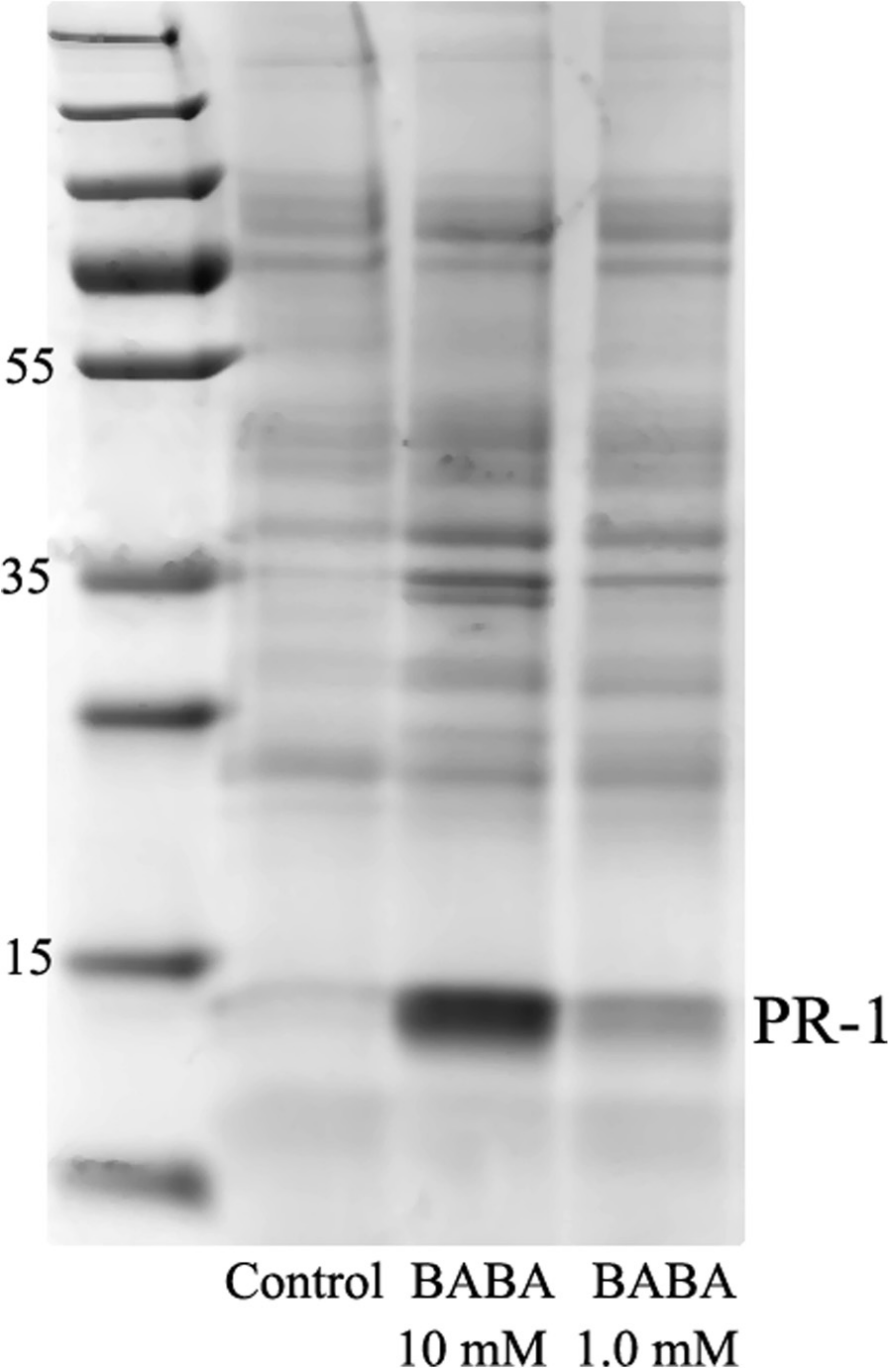
**SDS-PAGE of apoplast from Desiree leaves treated with BABA.** Separation on a SDS-PAGE gel, showing apoplast proteins extracted two days after treatment with BABA in cv. Desiree. From left: control plant treated with water, plant treated with 10 mM BABA and plant treated with 1 mM BABA. The ladder used was Page Ruler™ Plus Prestained Protein Ladder (SM1811, Fermentas).

The quality of the apoplast samples were checked by separation on SDS-polyacrylamide gels prior to protein identification by mass spectrometry (Figure 5). A thick band previously identified by us as PR-1 [35] was found after application of 10 mM BABA. A faint PR-1 band was seen after 1 mM BABA whereas no PR-1 band was detected in the water control.

In the apoplast secretome, we identified 24 proteins with altered abundance in potato after treatment with 1 mM BABA (Table 1; Table 5), compared to 91 proteins with 10 mM (Table 1; Table 6). Almost half of the 24 proteins affected by 1 mM BABA were affected in the same way by 10 mM BABA. Among these, six were up-regulated: two glucan endo-1, 3-beta-D-glucosidases (Q70BW9, G400012702), two endochitinases (G400008796, G400001528), pathogen-and wound-inducible antifungal protein CBP20 (PR-4; G400019437) and one annotated as a resistance gene-like (Q93YA6); and five down-regulated: 24 K germin (G400013010), kunitz trypsin inhibitor (G400027052), PAE (G401024140), beta-galactosidase (G402009228) and subtilisin-type protease (G400009947). Under both BABA conditions, there was a dose dependent response where 10 mM led to 1.4 to 11 fold higher protein abundance. The four most abundant secreted proteins affected by 10 mM BABA, were an Nb cell death marker (G400010131) with low sequence similarity to a Kunitz-type protease in *Arabidopsis* (At1G17860), a Sn-1 protein (G400011716), an acidic class II 1,3-beta-glucanase (PR-2) (Q8GUR4) and a pathogenesis-related leaf protein 6 (P04284) annotated as a basic PR-1 protein (PR1b1) in tobacco. Overall, proteins like thaumatin, glucanases, glucosidases, P69 proteins, peroxidases, PRp27 protein, pathogen-and wound-inducible antifungal protein CBP20 and chitinases, all belonging to pathogenesis-related (PR) protein families, were the most abundant among the proteins up-regulated by BABA.

**Table 5:** Proteins with differential abundance in the apoplast of potato leaves treated with 1 mM BABA

**Protein name**	**External IDs**	**log2 Fold change (peptide median)**^**2**^
1,3-beta-glucan glucanohydrolase	Q70BW9	20
Conserved gene of unknown function	P400042337	19
Acidic class II 1,3-beta-glucanase (Fragment)	Q8GUR3	17
Glucan endo-1,3-beta-D-glucosidase	P400022509	17
Resistance gene-like	Q93YA6	17
Basic 30 kDa endochitinase	P400015454	14
Leucine aminopeptidase 1, chloroplastic (DR57)	Q10712	13
Pathogen-and wound-inducible antifungal protein CBP20	P400033771	1.3
Glucan endo-1,3-beta-D-glucosidase	P400018563	1.2
Strictosidine synthase	P400030201	1.1
Endochitinase	P400002757	0.9
Miraculin	P400018006	-1.1
Kunitz trypsin inhibitor	P400046980	-16
Peptidyl-prolyl cis-trans isomerase	F4K2G0	-17
Heparanase-2	P400042675	-18
Thioredoxin	P400021964	-18
PAE	P400041742	-18
Peroxidase	P400026173	-18
Subtilisin-type protease	P400017566	-18
Beta-galactosidase	P400016282	-18
P69B protein	P400007007	-19
Leucine-rich repeat family protein	P400011041	-19
24 K germin	P400023053	-20
Major latex	P400046294	-21

Samples were analysed two days after treatment of the leaf canopy with BABA. Proteins with significantly changed abundance (FDR <0.1) are included in the table.

**Table 6:** Proteins with differential abundance in the apoplast of potato leaves treated with 10 mM BABA

**Protein name**	**External IDs**	**log2 Fold change (peptide median)**
Pathogenesis-related leaf protein 6 (P6) (PR protein)	P04284	23
Acidic class II 1,3-beta-glucanase (Fragment)	Q8GUR4	23
Nb cell death marker	P400017936	22
Sn-1 protein	P400020769	21
Cell wall peroxidase	P400048009	21
1,3-beta-glucan glucanohydrolase	Q70BW9	20
Resistance gene-like	Q93YA6	20
Citrate binding protein	Q8H9C1	20
Cytoplasmic aconitate hydratase	Q56WE8	20
Glucan endo-1,3-beta-D-glucosidase	P400022509	19
Acetylornithine deacetylase	P400030587	18
Transketolase, chloroplastic	P400038185	18
Beta-galactosidase	P400000661	17
Peptidyl-prolyl cis-trans isomerase	P400002952	14
Cationic peroxidase	P400022299	4.3
ADP-ribosylation factor 3	P400047760	3.8
Gene of unknown function	P400068931	3.8
Class III peroxidase	P400001015	3.8
Beta-1,3-glucanase, acidic	P400022914	3.8
Glycerophosphodiesterase	P400038623	3.4
Chlorophyll a-b binding protein 6A, chloroplastic	P400040595	3.3
Peroxidase	P400055404	3.0
Thaumatin	P400007581	2.9
Retinoid-inducible serine carboxypeptidase	P400000831	2.8
Glucan endo-1,3-beta-D-glucosidase	P400051976	2.7
AT5G66190 protein (Fragment)	B9DI26	2.7
Basic 30 kDa endochitinase	P400015454	2.6
Pathogen-and wound-inducible antifungal protein CBP20	P400033771	2.5
NtPRp27-like protein	Q84XQ4	2.5
Glucan endo-1,3-beta-D-glucosidase	Q42890	2.4
Glucan endo-1,3-beta-D-glucosidase	O82063	2.3
Putative glucan endo-1,3-beta-D-glucosidase	Q2HPL1	2.2
NtPRp27	P400011122	2.2
Glutathione reductase, cytosolic (GR) (GRase)	P48641	2.1
Endochitinase (Chitinase)	P400015232	2.0
Peroxidase	Q43774	2.0
P69E protein	P400007009	1.9
DUF26 domain-containing protein 1	P400063324	1.9
Cystatin	P400014844	1.8
Patatin 3	P400017707	1.7
Endochitinase (Chitinase)	P400002757	1.5
Peroxidase	P400043335	1.5
Acidic class II 1,3-beta-glucanase	P400018562	1.4
1,3-beta-D-glucan glucanohydrolase (Glucan endo-1,3-beta-glucosidase a)	Q70C53	1.4
STS14 protein	P400038079	1.4
Acidic endochitinase	P400056271	1.3
Lignin-forming anionic peroxidase (TOPA)	P11965	1.3
Glucan endo-1,3-beta-glucosidase	P400001406	1.1
Uncharacterized protein	K4ASJ5	1.1
Reticuline oxidase	P400031346	0.9
Aspartic proteinase nepenthesin-1	P400009908	-0.6
Kunitz-type proteinase inhibitor group B1 (Fragment)	Q58I49	-0.7
Conserved gene of unknown function	P400010730	-0.8
Catechol oxidase B, chloroplastic	P400051502	-0.8
24 K germin	P400023053	-0.8
Beta-hexosaminidase 1	P400054228	-0.9
Pectinesterase-2	P400049953	-1.1
Reticuline oxidase	P400042020	-1.1
Alpha-L-arabinofuranosidase	Q76LU4	-1.1
Acidic 27 kDa endochitinase	P400002758	-1.4
Phosphoesterase family protein	P400039390	-1.6
Kunitz trypsin inhibitor	P400046981	-1.9
Hydrolase, hydrolyzing O-glycosyl compounds	P400016965	-2.0
Peroxidase N	P400041612	-2.1
Zinc finger protein	P400004464	-16
H^+^transporting two-sector ATPase, alpha/beta subunit, central region	P400001921	-16
Periplasmic beta-glucosidase	P400016780	-16
Subtilisin-like protease	P400026165	-16
Kunitz trypsin inhibitor	P400046980	-16
Erg-1	P400022271	-16
Ferredoxin-dependent glutamate synthase 1	P400017124	-16
Glycolate oxidase	P400048099	-17
Cucumisin	P400010997	-17
Serine-threonine protein kinase, plant-type	P400029544	-17
Auxin-induced beta-glucosidase	P400039774	-18
ATP synthase subunit alpha, chloroplastic	P00823	-18
PAE	P400041742	-18
Cysteine protease inhibitor 1	P400017942	-18
Sn-1 protein	P400013688	-18
Salicylic acid-binding protein 2	P400001464	-18
Beta-galactosidase	P400016282	-18
Subtilisin-type protease	P400017566	-19
Hydrolase	P400031772	-19
Cellulase containing protein	P400015021	-20
Alpha-galactosidase/alpha-n-acetylgalactosaminidase	P400043893	-20
Beta-galactosidase	E3UVW7	-20
GDSL-like Lipase/Acylhydrolase family protein	P400011471	-20
Uncharacterized protein	K4CNZ8	-20
Receptor protein kinase CLAVATA1	P400020982	-20
Xyloglucan endotransglycosylase LeXET2	Q9FZ05	-21
Xylem serine proteinase 1	P400033261	-22

Samples were analysed two days after treatment of the leaf canopy with BABA. Proteins with significantly changed abundance (FDR < 0.1) are included in the table.

## Discussion

### Novel functional annotation and overall changes

In this paper we provide the potato research community with a more comprehensive functional annotation of the potato PGSC gene models and potato microarray probes (Agilent JHI *Solanum tuberosum* 60 k v1; Additional files 4 and 5). This new functional annotation was used for the interpretation of the BABA-IR response.

BABA at a concentration of 10 mM alters the expression of over 5000 transcripts and secretion of over 90 proteins to the apoplast. This is in contrast to the finding that only six transcripts (1 up- and 5 down-regulated) and 24 proteins (11 up- and 13 down-regulated) were affected by 1 mM BABA. This may explain the results from an earlier study by Liljeroth et al., 2010 [29], where 5 mM BABA was required to affect *P. infestans* infection rate, whereas BABA applied at concentrations below 2.5 mM failed to restrict *P. infestans* growth in the three tested potato cultivars. In agreement with this, we found that treatment of potato cv. Desiree with 10 mM BABA has a clear inhibitory effect of *P. infestans* infection. Whether the differences seen after treatment with 1 mM BABA, especially in the apoplast, lead to an increased resistance against other pathogens still has to be tested.

Furthermore, we can confirm the up-regulation of family members of proteinase inhibitor type-2:s, ubiquitin-conjugating proteins, glutathione S-transferases, lipoxygenases, histone H3.2 and a sesquiterpene synthase 2 previously identified by Li et al., 2009 [13] who reported 65 up-regulated transcripts based on cDNA-AFLPs after treatment with 4 mM BABA in potato.

The only up-regulated transcript by 1 mM BABA, a MutT/nudix domain protein, was also up-regulated by 10 mM BABA. Several MutT/nudix were also found to be up-regulated after BABA treatment in *Arabidopsis *[9]. This family is not well characterised functionally, but some members use their hydrolase activity to maintain cell homeostasis and have been implied to play a role in plant defence. For example, over-expression of AtNUDX2 resulted in increased pyrophosphatase activity toward ADP-ribose and increased tolerance to oxidative stress [36] and AtNUDX7 has been identified as an SA-independent factor in Enhanced Disease Susceptibility1 (EDS1)-controlled plant defence and programmed cell death [37]. In line with these observations, we found an ADP-ribose polymerase 2 and several other ADP-ribosylation factors as well as EDS1 to be transcriptionally up-regulated by 10 mM BABA in potato.

When comparing the transcripts and apoplastic protein abundance after 10 mM BABA treatment, regulation of 13 of the 70 proteins with peptides matching the predicted potato genome models overlapped. The samples are taken two days after treatment with BABA in both the transcript and protein analysis, but the overlap might have been greater if the RNA samples were taken earlier since there is an expected time lag between transcript and protein synthesis. There are also differences in transcript and protein stability. Another reason for these differences might be that protein abundance in the apoplast is at least partially regulated by protein transport and vesicle trafficking, and thus not directly dependent on transcript levels. Cytoplasmic membrane-bound vesicle was the most enriched GO term and several transcript encoding proteins in vesicle trafficking, such as clathrin assembly protein, Epsin-2 and PEN1 were all found to be between 2.5- and 11-fold up-regulated. The low overlap between transcript regulation and apoplast protein abundance confirms the value of combining the two methods.

### Immune responses and sterols

Several typical stress-response genes up-regulated by BABA in potato (e.g. *PR-1, PR-2, PAD4, thaumatin, aspartyl protease family protein*) have previously been found to be up-regulated in an *Arabidopsis* microarray study, where 46 genes in total were up-regulated about 3 days after treatment with 0.25 mM BABA [8]. In the present study, no known annotated R-genes and only a few other NBS-LRR proteins were up-regulated by BABA, which might indicate that BABA-IR is not mediated by increased expression of these genes through R-gene recognition and effector-triggered immunity (ETI). Instead most of the up-regulated genes and proteins affected by BABA were PR-proteins and components involved in hormone signalling and defence signalling such as PAMP triggered immunity, PTI. The up-regulated potato PEN1 homolog, which encodes membrane associated syntaxin, is involved in PTI, where it plays a part in the vesicle-based secretion system [38]. *EDS1*, *PAD4* and *SAG101*, genes implicated to play a part in non-host resistance [39], were also up-regulated by 10 mM BABA. However, none of them have been shown to be required for the defence against *P. infestans* in *Arabidopsis *[40-42]. Another gene among the four most up-regulated transcripts involved in stress response encodes osmotin (PR-5). This protein might play a role in BABA-IR to drought stress, as its abundance was increased in the leaf proteome of crabapple seedlings that were treated with BABA prior to drought stress exposure [43].

In a recent study, Kopischke et al. [44] reported that sterol balance plays a role in *Arabidopsis* non-host resistance to *P. infestans.* In their study, the *erp1* mutant lacking sterol ester accumulation experienced increased cell death. Oomycetes lack the ability to synthesize sterols, which are essential for their reproduction, and therefore depend on their hosts for sterol compounds [45]. The sterol biosynthesis within the mevalonate pathway was clearly down-regulated in the present study, whereas the biosynthesis of another branch of the pathway, namely sesquiterpene phytoalexin, was up-regulated (Figure 3). These results are in line with earlier findings in potato, where wound-induced sterol and steroid glycoalkaloid synthesis are suppressed in favour of sesquiterpenoid phytoalexin biosynthesis when potato tubers are exposed to elicitors or pathogens that induce HR [46,47]. This redirection of the isoprenoid biosynthetic pathway has been linked to changes in expression of *3-hydroxy-3 methylglutaryl-coenzyme A reductase* (HMGR) genes, where *hmg*2 and *hmg*3 are up-regulated whereas *hmg*1 is suppressed [48,49]. In the present study two HMGRs were up-regulated by 10 mM BABA. In a study conducted by Ros et al. [50], sesquiterpene synthase and vetispiradiene synthase were up-regulated after infection with *P. infestans* in two field-resistant cultivars, Bettina and Indira. It had earlier been shown that arachidonic acid, a fatty acid present in the lipids of oomycetes*,* can suppress steroid-glycoalkaloids [51] and act as an elicitor of sesquiterpene phytoalexins [52] with the involvement of LOX [53,54] in potato tubers. LOX is also among the 50 most up-regulated transcripts by 10 mM BABA in this study. However, BABA-IR in potato has been shown to require SA but not 9-LOX-derived oxylipins [14].

When comparing the present secretome data with that obtained from three genotypes, SW93-1015, Sarpo Mira (*P. infestans* incompatible) and Desiree (*P. infestans* compatible), 24 and 72 hpi with *P. infestans* (Ashfaq Ali, personal communication), patatin 3 (P400017707) increased in abundance after BABA treatment as well as infected SW93-1015 and Sarpo Mira, but not in susceptible Desiree. This is in line with previous findings that tobacco NTPAT1-3 is involved in cell death during HR [55], since HR are formed in SW93-1015 and Sarpo Mira but not Desiree. A role for patatin-like proteins in cotton HR has also been proposed [56].

### Amino acid metabolism

Earlier studies in *Arabidopsis* have revealed that BABA treatment alters amino acid composition and can be linked to an amino acid associated alternation of growth [10,11]. The BABA-mediated growth reduction and IR to *Pseudomonas syringae* pv. *tomato* DC3000 was compromised by treatment with L-glutamine [11]. The amino acid L-serine and the stress-responsive amino acids proline and asparagine were reported to be induced by BABA in *Arabidopsis*, whereas the aspartic acid content decreased (Singh et al., 2010). Transcripts involved in the proline biosynthesis were also observed after treatment with 10 mM BABA in the present study. Recently, a study in *Arabidopsis* revealed decreased abundance of several amino acids, including serine and glutamic acid, after treatment with potassium phosphite [57], and of aspartic acid after BABA treatment. However, whether a BABA-induced altered amino acid balance leads to changed leaf morphology in potato similar to those seen in *Arabidopsis*, remains to be investigated.

Another up-regulated transcript was arginine decarboxylase, which is involved in the biosynthesis of the polyamine putrescine by catalyzing the synthesis of agmatine from arginine. Polyamines are involved in a wide range of stress responses, partly as reactive oxygen scavengers [58]. Arginine decarboxylase has previously been reported to increase systemically in tobacco leaves after BABA treatment [59], which suggests a role for polyamines in BABA-IR.

### Hormones

SA has previously been shown to be important for basal defence against *P. infestans* and required for BABA-IR in the same potato pathosystem [14,60]. In the SA signalling pathway, transcripts of a phenlyalanine ammonia lyase (PAL) homolog were two fold up-regulated, whereas isochorismate synthase (ICS) did not change significantly. ICS, in contrast to PAL, is directly involved in the SA biosynthesis and has been shown to be up-regulated after BABA-treatment of *Arabidopsis *[8,9]. In contrast to SA, genes involved in JA, ET, GA and ABA biosynthesis were affected by BABA, and GO term analysis pointed to an enrichment of genes in GA biosynthesis.

In the present study, two splice variants of DELLA proteins, involved in GA signalling, were up-regulated. DELLA proteins play a role in resistance to necrotrophs and susceptibility to biotrophs by modulating the SA/JA signalling [61]. This modulation is accomplished by competitive binding of DELLAs to JAZ proteins, which prevents interaction between JAZ and MYC2, thus promoting MYC2-induced responses such as LOX [61]. Navarro et al. (2008) further suggested that GA might change this balance by degradation of DELLAs, thus promoting resistance to biotrophs and susceptibility to necrotrophs. Thus, it would be interesting to further investigate the role of DELLAs in BABA-IR in potato.

9-cis-epoxy-carotenoid dioxygenase 2 (NCED2), which is involved in the biosynthesis of, for example, ABA, was up-regulated. In a previous study in *Arabidopsis*, BABA treatment was shown to lead to the accumulation of several ABA responsive elements and it was suggested that crosstalk occurs between BABA and ABA signalling [9]. The ABA responsive transcription factors, identified as BABA-induced in *Arabidopsis* e.g. ABI1, 2 and 3, were not differentially expressed in BABA-treated potato. Instead, the only ABA-related transcription factor we could identify as differentially regulated, a bZIP, was repressed after BABA treatment.

Several genes related to BRs and auxins, which can affect plant development and cell wall formation, changed expression after treatment with BABA e.g. *TIR1* and several *auxin-response factors* (*ARFs*), but not ARF5, were down-regulated. Repression of auxin-related processes has repeatedly been observed in defence signalling. In *Arabidopsis*, Wang et al. [62] found that a number of auxin responsive genes were down-regulated after treatment with the SA analog benzothiadiazole S-methyl ester (BTH). With some exceptions, e.g. auxin-induced SAUR and auxin responsive factor 5 (ARF5), this is also true in our data. Furthermore, the over-expression of a rice IAA-amino synthetase, GH3, led to an enhanced disease resistance to the rice pathogen *Xanthomonas oryzae,* possibly due to down-regulation of expansins, leading to decreased loosening of the cell walls [62,63]. However, we see a clear up-regulation of several GH3 isoforms, but no significant change in expression of expansin transcripts in potato.

### Secreted proteins

Data obtained from the analysis of the apoplastic proteins revealed a high number of proteins related to defence and metabolism in the secretome of BABA-treated leaves. Several pathogenesis-related (PR) proteins were found to be highly abundant following BABA treatment. These proteins are used as markers for pathogen response and are induced by different biotic and abiotic stresses [64]. Among these were an acidic class II 1, 3-beta glucanase (PR-2) and thaumatin. PR-2, but not thaumatin, also showed increased abundance after BABA treatment of drought-stressed crabapple seedlings [43]. Another highly induced protein of less known biological function was the alginate-lyase motif containing citrate binding protein not present in the *Arabidopsis* genome [65]. The transcript encoding the citrate binding protein was also among the most up-regulated and has been described to be induced in potato after inoculation with *P. infestans *[50].

A cell wall peroxidase was highly induced after BABA treatment. These are known to be involved in cell wall strengthening following stress challenge and function by combatting degradation enzymes produced by pathogens [66]. Furthermore, peroxidases are involved in a number of defence-related responses such as HR, cross-linking of phenolics, and phytoalexin production. Peroxidases can be induced by different stresses and some plant defence activators including BABA [67]. A Snakin (Sn-1) protein was also highly up-regulated. Snakin proteins are known for their antimicrobial role in plant defence [68,69]. In addition, Sn-1 was recently found to play a role in leaf primary metabolism and cell wall composition in potato, suggesting a possible role in plant growth and development [70].

## Conclusions

Overall, at a concentration of BABA that gives reduced *Phytophthora* growth on potato, we find a global effect on the transcriptome and a large number of secreted proteins with increased abundance. Several processes related to classical plant hormones and amino acid metabolism seem to be affected and many known defence proteins are up-regulated. However, unlike the situation for Arabidopsis, ABA responsive genes seem not to be clearly regulated by BABA in potato. It is tempting to speculate that the strong down-regulation of the biosynthesis of sterols and steroid glucoalkaloids, in contrast to the up-regulation of important enzymes involved in sesquiterpene phytoalexin biosynthesis, could explain some of the BABA-IR against *P. infestans*.

Taken together, the clear direct effects of BABA treatment in potato might suggest a yield penalty. However, in our earlier study [29], the tuber yield was unaffected by weekly applications of BABA to field grown potatoes. The same result was reported for the tuber yield after repeated BABA treatments to potato grown in greenhouse conditions [71]. In fact, the tuber yield was improved in two of the cultivars used in the latter experiment. Thus, the individual genes and proteins, as well as general processes identified in this study, are possible candidates or markers for improved resistance without major influence on potato yield.

### Availability of supporting data

The microarray data was deposited in ArrayExpress, accession number: E-MTAB-1545.

## Abbreviations

ABA: Abscisic acid; ACN: Acetonitrile; AFLP: Amplified fragment length polymorphism; ANOVA: Analysis of variance; ARFs: Auxin-response factors; BABA: ß-aminobutyric acid; BR: Brassinosteroid; BAKs: Brassinosteroid insensitive 1-associated receptor kinase 1 s; cDNA: Complementary DNA; cv.: Cultivar; dpi: Days post infection; dH2O: Destilled water; DTT: Dithiothreitol; ETI: Effector-triggered immunity; ET: Ethylene; FDR: False discovery rate; GA: Gibberellic acid; GID: Gibberellic acid insensitive dwarf; Gene ontology terms: GO terms; HPLC: High-performance liquid chromatography; HR: Hypersensitive response; IR: Induced resistance; ISR: Induced systemic resistance; JA: Jasmonic acid; LRR: Leucine rich repeats; LTQ: Linear trap quadropole; MS: Mass spectrometry; mRNA: Messenger RNA; NBS: Nucleotide binding site; PTI: PAMP triggered immunity; PAMP: Pathogen-associated molecular patterns; PR protein: Pathogenesis-related protein; P. infestans: Phytophthora infestans; PAGE: Polyacrylamide gel electrophoresis; R-gene: Resistance gene; SA: Salicylic acid; SDS: Sodium dodecyl sulfate; SGR: Solanaceae genomics resource; SIMR: Stress-induced morphogenic response; SAR: Systemic acquired resistance; PGSC: The potato genome sequencing consortium; TIR: TOLL/interleukin-1 receptor.

## Competing interests

The authors declare that they have no competing interests.

## Authors’ contributions

TB, EOA, EL, EA planned the experiments and wrote the paper. LIM assisted in the lab experiment. SR and FL performed the proteomics and helped with the analysis. PH performed the microarray analysis. EOA and EPW helped with the bioinformatics analysis. DW and DJ designed and developed Parallel OrthoMCL and analysed the output data together with DDB. All authors read and approved the final manuscript.

## Supplementary Material

Additional file 1: Figure S1 Distribution of number of Gene Ontology terms. The number GO terms per probe of the Agilent JHI *Solanum tuberosum* 60 k v1 microarray annotated using either Parallel-OrthoMCL across 26 plant genomes or the Solanaceae Genomics Resource annotation. Click here for file

Additional file 2: Table S1 Complete list of enriched GO terms. Significant differentially expressed genes after 10 mM BABA treatment were analysed by GOEast and GO terms with p >0.1 were considered significant. Click here for file

Additional file 3: Table S2 Significant differentially expressed transcripts. A full list of significant differentially expressed transcripts (adj. p-value <0.05) in potato leaves following treatment with 10 mM BABA. Samples were analysed two days after treatment of the leaf canopy with BABA. Click here for file

Additional file 4 **Text file 1.** Extended gene ontology terms for microarray probes. Extended gene ontology terms for the custom-made Agilent expression array (JHI *Solanum tuberosum* 60 k v1). The file is formatted for use in GOEast. Click here for file

Additional file 5 **Text file 2.** Extended gene ontology terms for PGSC gene models v.3.4. Extended gene ontology terms for the PGSC gene models. The file is formatted for use in GOEast. Click here for file
