# Supplementary figures and images for: Proteomics and transcriptomics of the BABA-induced resistance response in potato using a novel functional annotation approach
Source: BMC Genomics. 2014 Apr 28;15:315. doi: 10.1186/1471-2164-15-315 (PMC4234511; doi:10.1186/1471-2164-15-315)

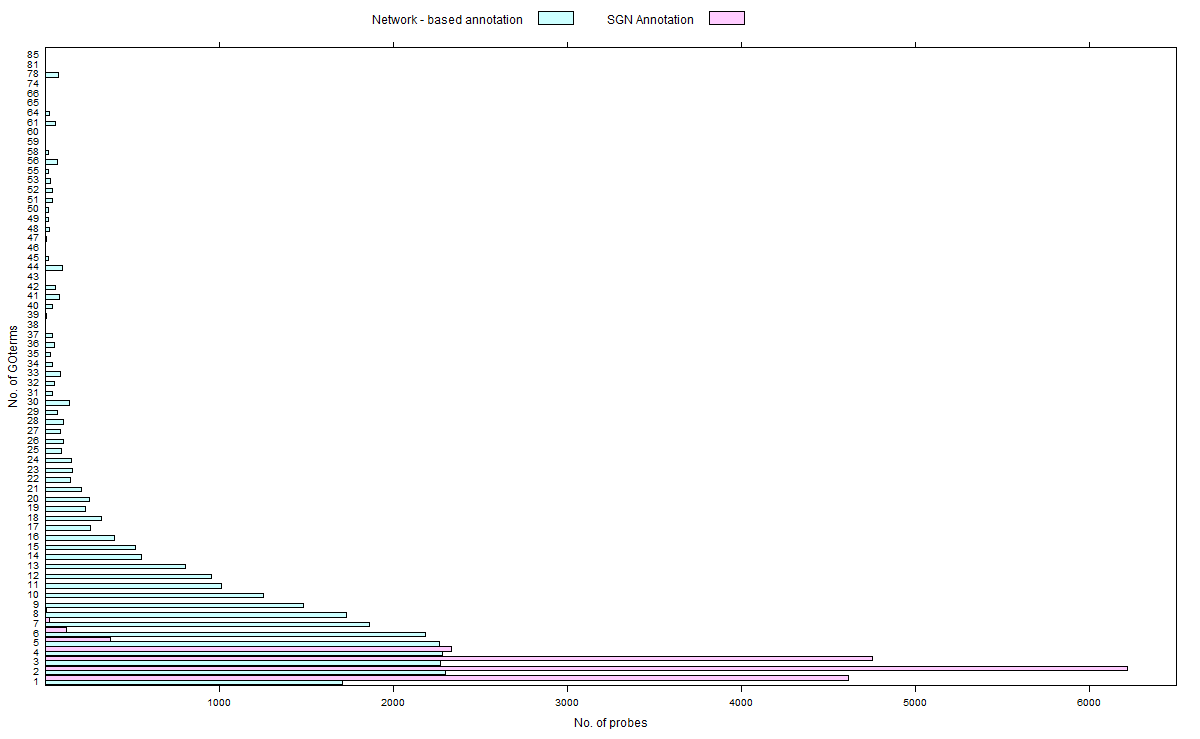

Supplement: Additional file 1: Figure S1 — Distribution of number of Gene Ontology terms. The number GO terms per probe of the Agilent JHI Solanum tuberosum 60 k v1 microarray annotated using either Parallel-OrthoMCL across 26 plant genomes or the Solanaceae Genomics Resource annotation. [file 1471-2164-15-315-S1.tiff]
